# Elevated histone deacetylase 10 expression promotes the progression of clear cell renal cell carcinoma by Notch-1-PTEN signaling axis

**DOI:** 10.1007/s12672-024-01018-9

**Published:** 2024-05-11

**Authors:** Bin Zheng, Xue Jiang, Yaqing Liu, Fajuan Cheng, Yiming Zhang, Chengtao Niu, Zixiang Cong, Zhihong Niu, Wei He

**Affiliations:** 1 Department of Urology, Shandong Provincial Hospital, Shandong University, Jinan, 250021 Shandong China; 2 Department of Urology, Shandong Provincial Hospital Affiliated to Shandong First Medical University, Jinan, 250021 Shandong China; 3 Department of Nephrology, Shandong Provincial Hospital, Shandong University, Jinan, 250021 Shandong China

**Keywords:** Histone deacetylase, Renal cell carcinoma, Prognosis, Biological function

## Abstract

**Background:**

Clear cell renal cell carcinoma (ccRCC), the most common pathological subtype of kidney cancer, accounts for approximately 70% to 80% of all cases. Histone deacetylase 10 (HDAC10) belongs to the HDAC class IIb subgroup, one of the histone deacetylases (HDAC) family. Previous studies suggest that HDAC10 may regulate the development of multiple tumor types. The specific molecular mechanisms employed by HDAC10 in the etiology of ccRCC still need to be discovered.

**Methods:**

The analysis included examining HDAC10 expression levels and their clinical importance within a cohort of inpatients and ccRCC patients documented in the Tumor Genome Atlas (TCGA). Moreover, the biological functions and underlying molecular mechanisms of HDAC10 were investigated.

**Results:**

HDAC10 showed increased expression in ccRCC tumor tissues. Subsequent analysis revealed overexpression of HDAC10 was associated with advanced clinical phenotype and unfavorable prognosis. The absence of HDAC10 significantly decreased ccRCC cell proliferation and migration capabilities. Mechanistic research suggests that HDAC10 may promote RCC development by activating the Notch-1 pathway and downregulating PTEN expression levels.

**Conclusion:**

In summary, HDAC10 can modulate critical biological processes in ccRCC, including proliferation, migration, and apoptosis. Notably, the Notch-1 pathway and PTEN serve as crucial signaling pathways and target genes through which HDAC10 regulates the progression of ccRCC. These findings offer a novel outlook for ccRCC treatment.

**Supplementary Information:**

The online version contains supplementary material available at 10.1007/s12672-024-01018-9.

## Introduction

Renal cell carcinoma (RCC) is the 14th most common cancer overall [1]. The incidence of kidney cancer is increasing in Asian countries. RCC is more prevalent in northern and urban regions of China [2]. The most predominant subtype is clear cell renal cell carcinoma (ccRCC), accounting for 70% to 80%of cases [3]. Although the pathogenesis of RCC is not fully understood, recent data confirm that RCC is fundamentally a metabolic disease. Indeed, many studies have shown that altered metabolism is involved in the development of RCC and that many of the genes altered in this tumour play an important role in controlling cellular metabolic activity. In addition, the introduction of high-throughput histology techniques has not only provided detailed molecular characterization of RCC but has also identified biomarkers, allowing for more accurate prognostic stratification [4]. About 25–30% of individuals diagnosed with ccRCC present with locally advanced disease or distant metastases, and renal cancer commonly manifests without apparent clinical symptoms. Despite undergoing treatment for localized tumors, around one-third of patients face recurrence, resulting in an unfavorable prognosis [5].

There are 18 distinct histone deacetylases (HDACs) in humans, categorized into four classes according to sequence similarity [6]. Aside from their direct role in acetylation modifications, HDACs can also influence post-translational modifications and regulate gene transcription by modulating the interactions between DNA and histones [7, 8]. In 2002, the discovery of HDAC10 was reported by Fischer et al. [9], Tong et al. [10], Kao et al. [11], and Guardiola et al. [12]. HDAC10 is recognized as an enzyme with a dual function, exhibiting both lysine acetyltransferase and lysine deacetylase activities [13]. Research studies have demonstrated that HDAC10 contributes to tumor progression by exerting its epigenetic function and modulating distinct molecules and signaling pathways [14]. Previous studies have identified HDAC10 as an element component gene in clinical prediction models constructed on the basis of the HDACs family of genes, indicating that HDAC10 could serve as a potential molecule for ccRCC prognosis [15]. Nonetheless, the precise molecular mechanisms underlying the regulation of RCC's biological behavior, encompassing proliferation, migration, and invasion, by HDAC10 remain unexplored in previous studies.

This study aimed to investigate the predictive significance of HDAC10 in ccRCC and elucidate its underlying mechanisms. The findings demonstrated that HDAC10 could be a prognostic factor in patients with ccRCC. Mechanistic investigations have shown that HDAC10 plays a crucial role in regulating the progression of ccRCC by modulating the Notch-1 pathway and PTEN expression levels. Therefore, further research is warranted to explore HDAC10 as a promising diagnostic marker for patients with ccRCC.

## Materials and methods

### Bioinformatics analysis

TNM stage, clinical stage, overall survival (OS) data, and HDAC10 in RCC (KIRC) expression levels in the organization were obtained from the TCGA database (https://xenabrowser.net/heatmap/). Gene enrichment analysis (GSEA) was conducted to identify the signaling pathways implicated in the pathogenesis of ccRCC under conditions of elevated HDAC10 expression.

### Patient samples and cell lines

This study was approved by the Medical Ethical Committee of Shandong Provincial Hospital and written informed consent in accordance with the Declaration of Helsinki was obtained from each patient. The paraffin-embedded tissue of the patient in the study was re-embedded into new blocks for Immunohistochemical staining. Pathological specimens and clinicopathological characteristics were collected, and all samples were anonymous. Normal human embryonic kidney cell lines HEK-293 and human renal cell cancer cell lines 786-O, 769-P, Caki-1, Caki-2, OS-RC-2, and A498 were sourced from Procell. Cells were cultured in a medium supplemented with 10% fetal bovine serum (EXcellBio, Shanghai) and a penicillin/streptomycin mixture (Solepol Technology Co, Ltd, Beijing). The cells were incubated in a carbon dioxide incubator.

### Immunohistochemical (IHC) staining

Immunohistochemistry (IHC) was performed using standard immunoperoxidase staining protocols with examination of the specimens at 200 × and 400 × magnification. Two observers independently analyzed the staining results and calculated the corresponding IHC score. Rabbit antibodies against HDAC10 (Proteintech, USA) were used as the primary antibodies, and HRP-labeled goat anti-rabbit IgG (Xavier Biotechnology Ltd, Wuhan) was utilized as the secondary antibody.

### Lentiviral vector construction and cell transfection

Three specific sequences of HDAC10 were targeted using three shRNAs, designated as HDAC10 shRNA1: 5'-GATCCCGCCGGATATCACATTGGTTCTCTCGAG- AGAACCAATGTGATATCCGGCTTTTTGGAT -3'; HDAC10 shRNA2: 5'-GATCCCGCAGGTGAACAGTGGTATAGCCTCGAG-GCTATACCACTGTTCACCTGCTTTTTGGAT-3'; HDAC10 shRNA3: 5'-GATCCCGGCACCTGAACAGTGGTATAGCTCGAG-CTATACCACTGTTCACCTGCCTTTTTGGAT-3'. The sequences were forwarded to Genechem, where the lentiviral vector was constructed by Genechem Packaging. Afterward, the lentiviral vector was utilized to transduce the cells, and the downregulation of HDAC10 expression was verified by qRT-PCR at the RNA level, as described in the subsequent section.

### qRT-PCR

TRNzol universal total RNA extraction reagent (Beijing Tiangen Biochemical Technology Co., LTD.) was used to extract total RNA according to the manufacturer's scheme. Reverse transcription of RNA was performed according to the instructions using FastQuant cDNA first strand synthesis kit (KR116). Then qRT-PCR was performed, and the results were analyzed by 2^−ΔΔCt^. Primers used for PCR amplification are shown in Supplementary Table 1.

### Western blotting

Total protein was extracted initially, followed by determining the protein concentration using the BCA method. The protein solution sample was mixed with 5 × SDS-PAGE protein loading buffer at a 4:1 ratio and shaken well. The mixture was loaded onto the SDS-PAGE gel for electrophoresis and then transferred to the PVDF membrane. The membrane was incubated with HDAC10 (Proteintech, USA), GAPDH (Zhongshan Jinqiao, Beijing), actin (Zhongshan Jinqiao, Beijing), and other primary antibodies overnight. Afterward, the PVDF membrane was washed with TBST and incubated with the corresponding secondary antibody for 1 h. Finally, the PVDF membrane was exposed using an exposure solution.

### Colony formation assay

A cell concentration of 1500 cells/well was added to a 6 cm petri dish. The growth medium was replaced every three days, and the cells were cultured for approximately ten days. After that, the medium was removed, cells were fixed with 4% paraformaldehyde for 30 min, and stained with 1 ml of 0.1% crystal violet solution per well for 10–30 min.

### Cell proliferation assay

Cell proliferation was assessed using an MTT assay kit (Seville Biotech Ltd., Wuhan). A cell density of 10^5^ cells per well was inoculated onto a 96-well plate. The cells were gently agitated, adding 20 μl of 5 mg/ml MTT solution to each well. The plate was then incubated in the incubator for 4 h. Cell proliferation was assessed by measuring the absorbance at 570 nm, and the resulting absorbance readings at different time points were used to construct cell proliferation curves.

### Migration assays

The undersurface of the Transwell system (24-well plate, Corning Corporation) was coated with 500μL medium containing 10% serum. The upper chamber was loaded with 300μL of cells in a serum-free medium. Following a 24-h incubation, the cells in the Transwell chamber were fixed in a plate containing a paraformaldehyde solution for 30 min. Next, the cells were stained with a 0.1% crystal violet solution for 10 min. Images were captured and analyzed using fluorescence microscopy, with three high-power fields chosen randomly for cell counting.

### Cell cycle and apoptosis

After trypsinization, Cells were harvested and subjected to staining using the Cell Cycle staining kit (Hangzhou Unitech Biotech Ltd) per the manufacturer's instructions. Before staining take care to incubate the cells at 37 °C away from light for 30 min. Annexin V-APC/7-AAD Apoptosis Kit (Hangzhou Unitech Biotech Ltd) was also used for apoptosis analysis. Flow cytometry was then employed to determine the cell cycle distribution and measure apoptosis.

### Xenograft tumor model in vivo

Ten male BALB/c nude mice, aged 4–6 months, were procured from the Experimental Shopping Center of Shandong University. After one week of acclimatization, the ten nude mice were randomly assigned to the shNC and shHDAC10-2 groups. The subcutaneous xenograft tumor model was established by injecting 0.2 ml of A498 cell suspension, transfected with shNC and shHDAC10-2, respectively, into the subcutaneous axillary tissue of nude mice (1 × 10^7^ cells per injection). Once the tumors became visible, their longitudinal and transverse diameters were measured and recorded using vernier calipers, and the average measurements were calculated for each group. All procedures were approved by the Animal Care and Use Committee at Shandong Provincial Hospital.

### Statistical analysis

Statistical analyses were performed using Graphpad Prism 8.0 (GraphPad Software, San Diego, CA). All quantitative data are the mean ± standard deviation of three independent experiments. One-way ANOVA was used to assess the relationship between variables. Kaplan–Meier analysis was performed for survival curves. P < 0.05 differences were statistically significant (*P < 0.05, **P < 0.01, ***P < 0.001, ****P < 0.0001).

## Results

### HDAC10 is upregulated and predicts an unfavorable prognosis in ccRCC

**Fig. 1:**
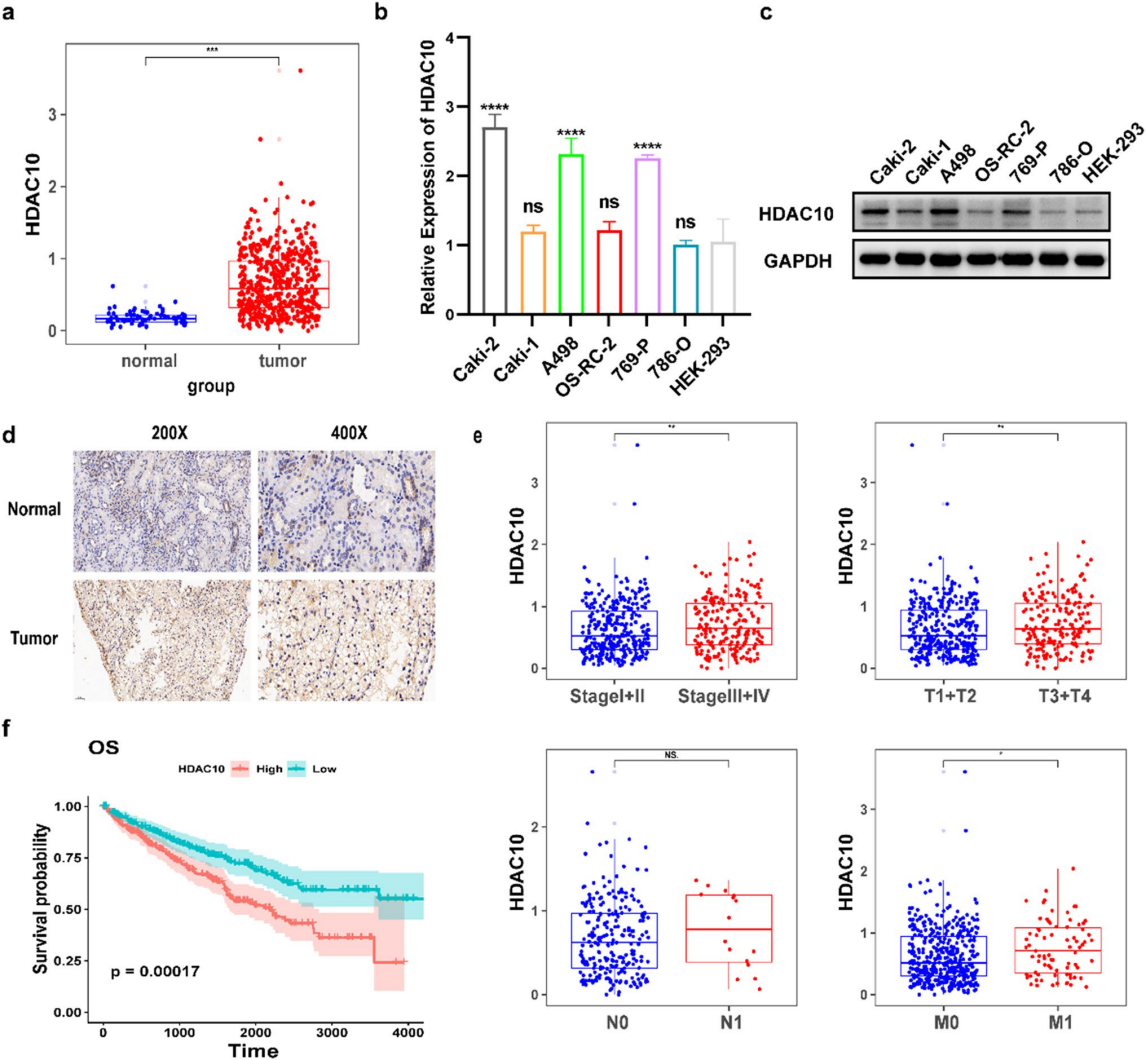
HDAC10 expression was upregulated in ccRCC and associated with poor prognosis **a** Analysis of HDAC10 mRNA expression in both normal renal cell lines and RCC cell lines. **b** Validation of HDAC10 mRNA expression levels in HEK-293 cells and tumor tissue using qRT-PCR. **c **Western blot showed HDAC10 protein expression in HEK-293 cells and tumor tissues. **d** Immunostaining analysis of HDAC10 expression in ccRCC tissues. Images were captured at magnifications of 200X and 400X. **e** Analysis of the TCGA-KIRC dataset revealed that HDAC10 overexpression was correlated with higher pathological T-stage, distant metastasis, and advanced clinical stage, while no significant association was observed with lymph node metastasis. **f** Kaplan–Meier survival analysis was performed on the OS of patients in the low HDAC10 mRNA level group (n = 266) and the high HDAC10 mRNA level group (n = 264). Critical values used for grouping were determined based on the mean of HDAC10 mRNA levels. (*P < 0.05; **P < 0.01; ***P < 0.001; ****P < 0.0001). ccRCC: clear cell renal cell carcinoma; KIRC: Kidney renal clear cell carcinoma; TCGA: The Cancer Genome Atlas database

To validate the expression levels of HDAC10, we initially analyzed the RNA-seq dataset of patients with ccRCC obtained from the TCGA (The Cancer Genome Atlas) database. These analyses revealed a significant upregulation of HDAC10 expression in ccRCC tissues (Fig. 1a).

Subsequently, we performed qRT-PCR analysis to validate the upregulation of HDAC10 expression in ccRCC cells, compared to the embryonic kidney cell line HEK-293 (Fig. 1b). Furthermore, western blotting analysis confirmed the upregulation of HDAC10 protein expression in ccRCC cell lines (Fig. 1c). Moreover, immunohistochemistry performed on tissue samples collected from our hospital revealed that HDAC10 exhibited weak or negative staining in paraneoplastic tissues while demonstrating robust and predominantly nuclear localization in ccRCC tissues (Fig. 1d). We utilized the data from the TCGA-KIRC dataset to assess the correlation between HDAC10 transcript levels and clinicopathological characteristics. The results are presented in Fig. 1e. Elevated HDAC10 expression significantly correlated with advanced pathological T-stage and metastasis but not with lymph node metastasis. To assess the prognostic significance of HDAC10, Kaplan–Meier analysis was performed using the TCGA-KIRC dataset. The results demonstrated a substantial association between high levels of HDAC10 and poor overall survival (OS) (Fig. 1f). These findings suggested that HDAC10 played an essential role in the progression of ccRCC.

### Inhibition of HDAC10 expression suppresses cell proliferation, migration, apoptosis, and cell cycle in ccRCC in vitro

**Fig. 2:**
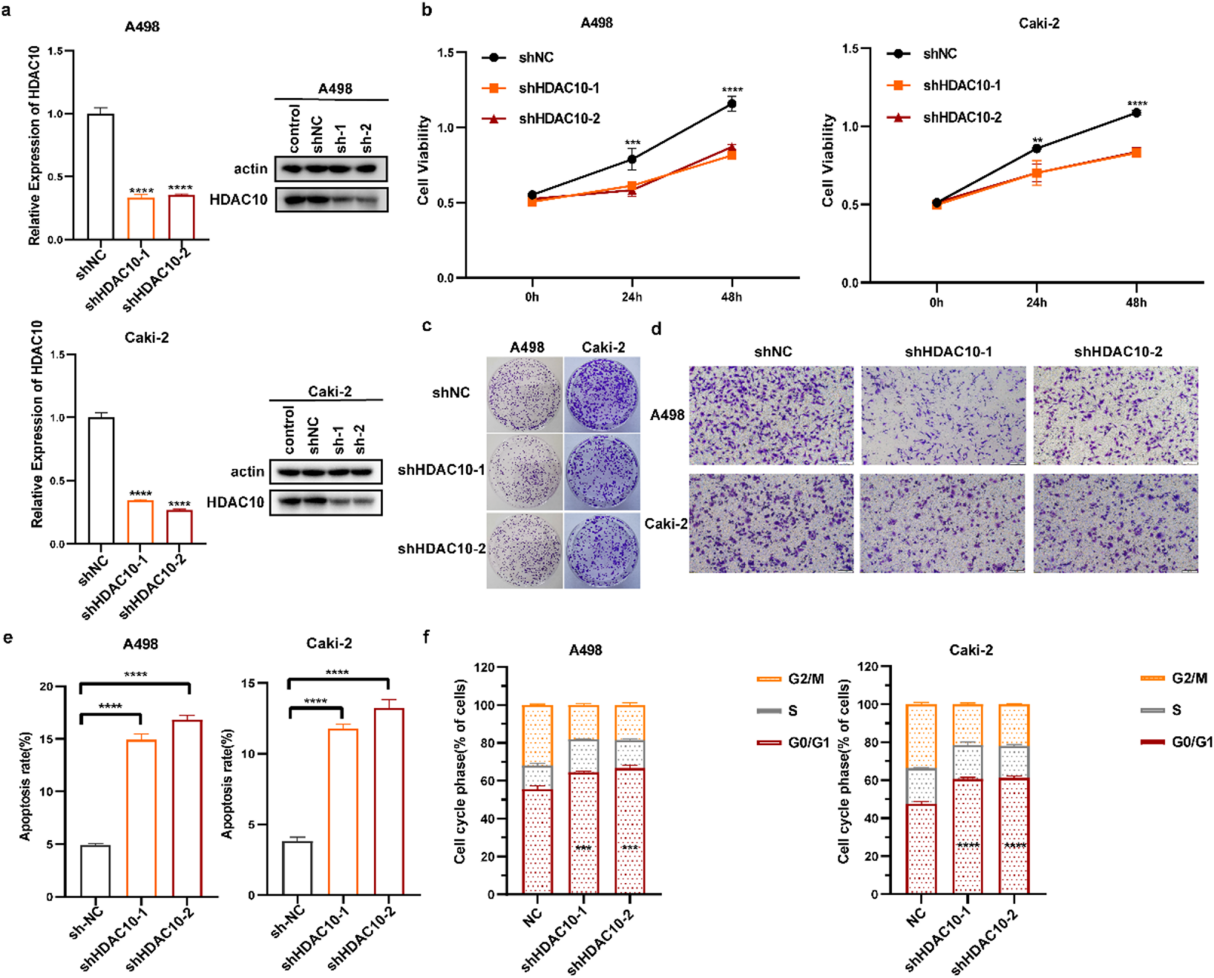
HDAC10 regulated cell proliferation, migration, apoptosis, and cell cycle in ccRCC in vitro **a** Validation of HDAC10 mRNA and protein knockdown in ccRCC. **b** MTT assay demonstrated the significant inhibitory effect of shHDAC10 on the proliferation of A498 and Caki-2 cells. **c** Colony formation test confirmed that the number of colonies decreased significantly after HDAC10 gene knockout. **d** Cell migration assay revealed a significant inhibition of cell migration by shHDAC10 (magnification 100x). **e** Increased apoptosis was observed treated with shHDAC10. **f** shHDAC10 induced a significant reduction in G2/M phase cells and an increase in G0/G1phase cells. (**P < 0.01; ***P < 0.001, ****P < 0.0001). ccRCC: clear cell renal cell carcinoma

qRT-PCR and Western blot analyses were performed to evaluate the expression levels of HDAC10 in ccRCC cells following HDAC10 knockdown. We found that the level of HDAC10 was significantly decreased in the shHDAC10 groups (Fig. 2a). We conducted MTT and Transwell assays to investigate the impact of HDAC10 silencing on the proliferation and migration of ccRCC. HDAC10 knockdown caused a significant decrease in the cell proliferation capacity of A498 and Caki-2 cell lines, as demonstrated by the MTT assay (Fig. 2b).

Additionally, the colony formation assays revealed a significant reduction in colony formation when HDAC10 was knocked down (Fig. 2c). The Transwell assays showed a noteworthy decrease in the migratory capacity of ccRCC cells after HDAC10 knockdown, as depicted in Fig. 2d. Subsequently, we evaluated alterations in the cell cycle and apoptosis through flow cytometry analysis after HDAC10 knockdown. The results revealed a significant increase in apoptosis in the HDAC10-knockdown cells. (Fig. 2e Supplementary Fig. 1a). Regarding cell cycle alterations, HDAC10 depletion induced an obvious increment of cell proportion in the G0/G1 phase (Fig. 2f Supplementary Fig. 1b). These experiments conclusively showed that downregulating HDAC10 expression might suppress ccRCC progression by regulating cell proliferation, migration, apoptosis, and cell cycle.

### The silencing of HDAC10 impedes the growth of ccRCC cells in vivo

**Fig. 3:**
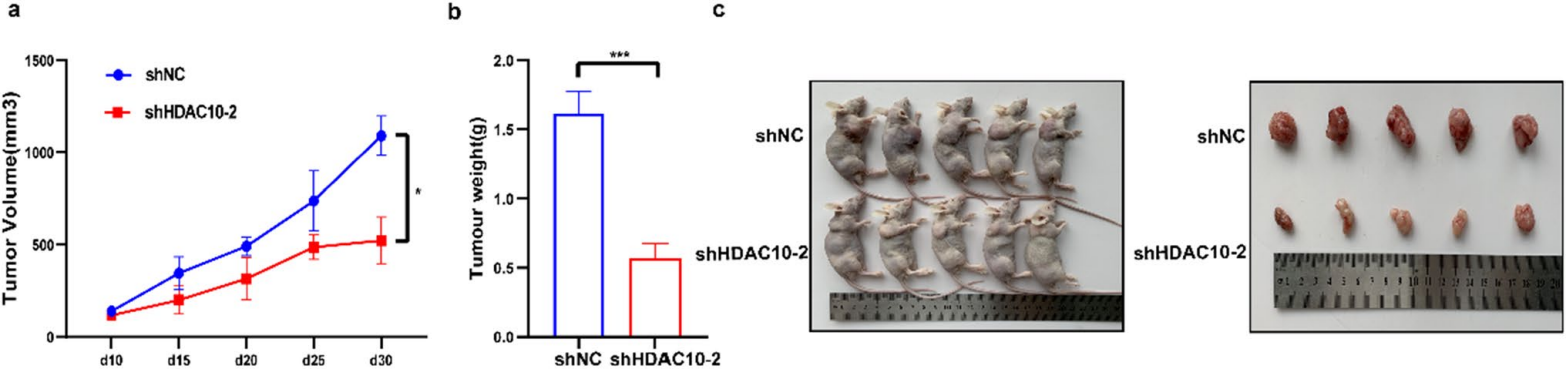
In vitro, knockdown of HDAC10 inhibited ccRCC tumour progression **a** Growth curves of xenograft experiments. **b** Tumour weight in xenograft experiments. **c** Pictures of nude mice and xenograft tumours. (*P < 0.05, ***P < 0.001). ccRCC: clear cell renal cell carcinoma

To examine the impact of HDAC10 knockdown on tumorigenesis, we established a xenograft model in which A498 cells transfected with shNC and shHDAC10-2 were inoculated subcutaneously in nude mice. The xenograft experiments demonstrated a significant decrease in growth rate (Fig. 3a), weight (Fig. 3b), and volume (Fig. 3c) of mouse xenograft tumors developed from shHDAC10 cells.

### HDAC10 promotes ccRCC tumorigenesis by regulating the Notch-1-PTEN signaling axis

**Fig. 4:**
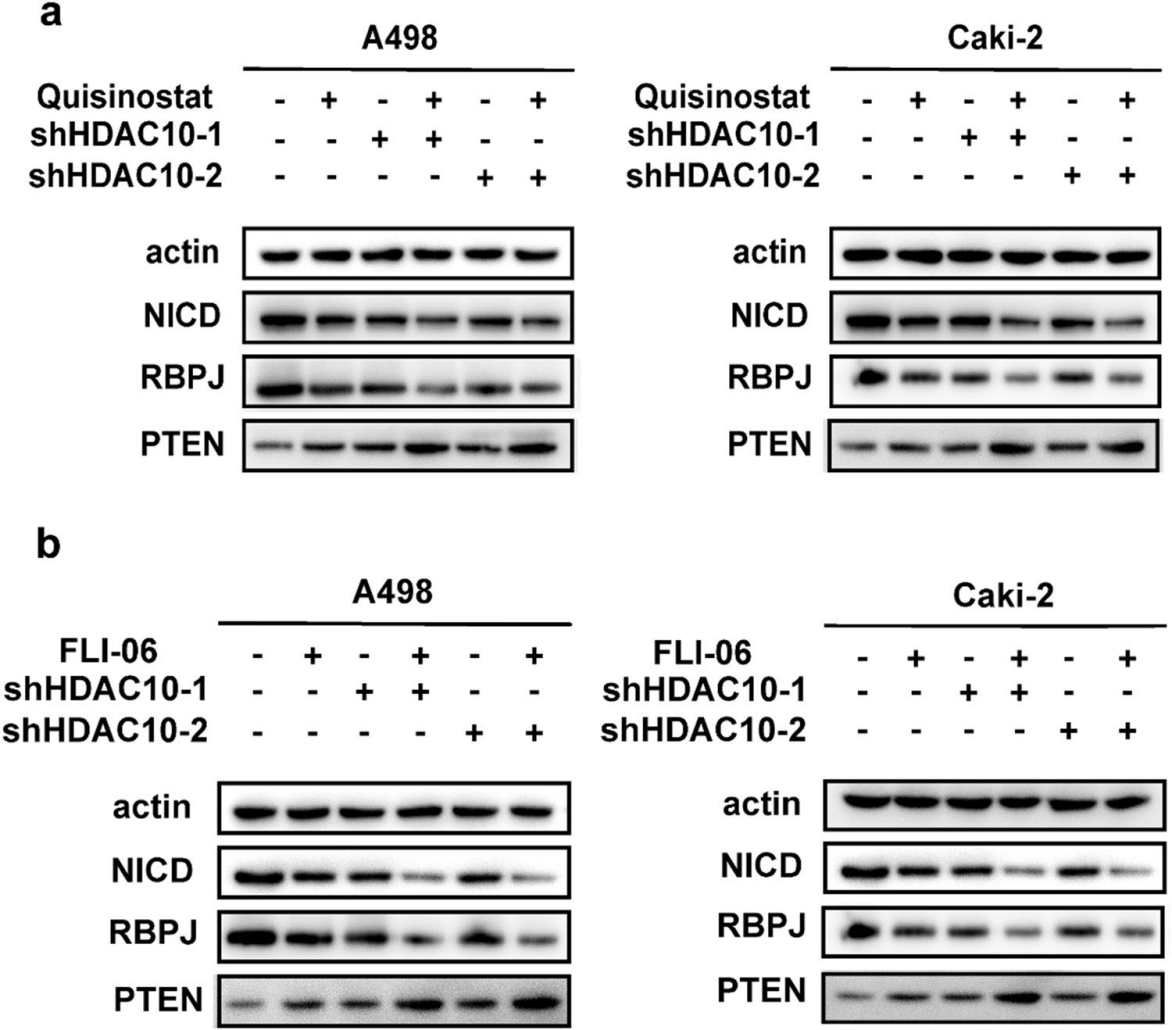
HDAC10 promoted the progression of ccRCC by regulating the Notch-1-PTEN signaling axis **a** Knockdown of HDAC10 expression levels and use of Quisinostat, an HDAC10 inhibitor, inhibited the Notch pathway and upregulated PTEN expression in A498 and Caki-2 cell lines. **b** The application of FLI-06 significantly elevated the expression levels of PTEN compared to the control group. (*P < 0.05, **P < 0.01, ***P < 0.001). ccRCC: clear cell renal cell carcinoma

The involvement of the Notch-1 pathway in the development of RCC and its significant role has been previously reported [16]. GSEA analysis revealed a positive modulation of Notch signaling pathways by HDAC10 (Supplementary Fig. 2a). Previous studies have indicated that aberrant expression of key proteins in the Notch-1 pathway could influence PTEN expression, thereby affecting tumor development [17]. The correlation between PTEN and RBPJ, a vital protein of the Notch-1 pathway, was also confirmed using the GEPIA (GEPIA (Gene Expression Profiling Interactive Analysis) (cancer-pku. cn)) database (Supplementary Fig. 2b). Therefore, we initially evaluated the expression of essential proteins involved in the Notch-1 pathway and PTEN through Western Blotting. Knockdown of HDAC10 in ccRCC cells caused a pronounced reduction in Notch Intracellular Domain (NICD) and recombination signal binding protein for immunoglobulin kappa J region (RBPJ) expression levels while a notable increase in PTEN levels, as shown in Fig. 4a. Subsequently, ccRCC cells were treated with the HDAC10 inhibitor Quisinostat, and the expression of downstream proteins in the Notch-1 signaling pathway and PTEN-related proteins was assessed by Western blotting. The data presented in Fig. 4a showed that treatment with Quisinostat increased the PTEN expression while significantly reducing the expression levels of NICD and RBPJ. These findings indicated that the acetylase activity of HDAC10 played a crucial role in regulating the expression of key components of the downstream Notch signaling pathway, including PTEN.

Subsequently, we introduced FLI-06, a known inhibitor of the Notch-1 signaling pathway. This result revealed a further elevation in PTEN expression levels (Fig. 4b). These findings revealed that HDAC10 might modulate the expression of PTEN by influencing the activation of the Notch-1 pathway, thereby promoting the progression of ccRCC.

## Discussion

In the last decade, many studies have shown that abnormalities in lipid metabolism play a crucial role in RCC, revealing the potential role of lipidomics in diagnostic and therapeutic studies. Recently, comprehensive molecular profiling studies have shed new light on the pathophysiology of ccRCC, which is widely regarded as a metabolic disease. In particular, it has been shown that alterations in specific intermediates involved in the tricarboxylic acid (TCA) cycle, glutamine or pentose phosphate (PPP) pathways are associated with a poorer prognosis. In recent years, their potential for biomarker detection and therapeutic targeting studies has been identified by integrating their role in ccRCC metabolism [18–21].

Several studies have shown that malignant transformation is associated with increased glycolytic flux as well as anaerobic and aerobic cellular lactate excretion. In addition, grade-dependent metabolic reprogramming has been found to occur in ccRCC tissues through the use of proteomic approaches and, more recently, combined proteomic and metabolomic analyses, with the Warburg effect being relatively more prominent in higher grade tumours. Renal cell carcinoma is fundamentally a metabolic disease [22–24]. In particular, sugars produced in the upper part of glycolysis are diverted to the pentose phosphate pathway, whereas triose phosphate produced in the lower part is diverted to the Krebs cycle or one-carbon metabolism. In this case, mitochondrial bioenergetics and oxidative phosphorylation processes are impaired in ccRCC, facilitating increased glucose utilisation via the pentose phosphate pathway. The phospholipid-binding protein Annexin A3 (AnxA3), a negative regulator of adipocyte differentiation, is down-regulated in RCC and shows different expression patterns for both 36 and 33 kDa isoforms. Silencing of 36 kDa AnxA3 in ccRCC cells increased adipogenic medium-induced lipid storage [4, 22, 25, 26].

Despite improvements in the five-year survival rate for ccRCC, the overall prognosis remains unfavorable for patients [27]. While significant progress has been made in comprehending the molecular mechanisms involved in renal cell carcinogenesis through molecular biology studies, exploring novel molecular targets and indicators that can improve the diagnosis and treatment of this condition remains imperative. In this scenario, it has been shown that histone deacetylases have a role as a regulator of cancer cell metabolism, However, it is not well defined in renal cancer.

HDAC10 plays a pivotal role in regulating tumorigenesis and metastasis [15]. The study aims to elucidate the biological function of HDAC10 in ccRCC. We comprehensively evaluated HDAC10 expression in ccRCC by analyzing cancer cell lines and clinical samples, revealing a substantial upregulation of HDAC10 in ccRCC. Moreover, we identified robust associations between HDAC10 overexpression and advanced pathological T-stage, clinical stage, and distant metastasis. Subsequently, we utilized Kaplan–Meier analysis to assess the prognostic significance of HDAC10 in ccRCC patients. The results demonstrated that overexpression of HDAC10 is predictive of an unfavorable prognosis. We investigated the effect of HDAC10 on the proliferation and migration of ccRCC cells by downregulating its expression levels and confirming the knockdown efficiency. Downregulation of HDAC10 expression caused a significant inhibition of ccRCC cell proliferation, as evaluated by the MTT assay.

Furthermore, flow cytometry analysis demonstrated a substantial increase in apoptosis in ccRCC cells following HDAC10 knockdown. The knockdown of HDAC10 causes ccRCC cell cycle arrest in the G0/G1 phase, which affects cell proliferation and was analyzed by flow cytometry. The Transwell assay has revealed that the migratory ability of ccRCC cells was significantly reduced after HDAC10 knockdown.

The Notch-1 pathway was initially described by the American geneticist Morgan [28]. The Notch-1 pathway is a highly conserved signaling pathway. It is involved in various aspects of tumorigenesis, such as cell proliferation and cell differentiation [29]. The importance of the Notch-1 signaling system in controlling tumor biology is evident through its involvement in processes such as epithelial-mesenchymal transition (EMT), drug resistance, cell proliferation, differentiation, apoptosis, and metastasis [30]. Somatic mutations of the Notch gene have been identified in T-ALL, CLL, NSCLC, and other malignancies [31, 32]. Additionally, dysregulation of downstream signaling within the wild-type Notch pathway has been documented in various cancers, including breast, and prostate cancer, among others [33, 34]. This dysregulation leads to the cleavage of the metalloproteinase TACE, resulting in the formation of NICD. Subsequently, NICD can bind to the RBPJ and influence tumor development [35]. The Notch-1 pathway plays an important role in tumor development by downregulating the expression of the PTEN in specific types of tumors, such as breast cancer and T-cell leukemia [36, 37].

We were considering the critical role of the Notch-1 signaling pathway in gene expression regulation and the contribution of different HDAC families in tumorigenesis via this pathway. Following HDAC10 gene knockout, the expression levels of NICD and RBPJ in ccRCC cells were significantly reduced. To assess the impact of HDAC10's deacetylation activity, we treated ccRCC cells with Quisinostat, an HDAC10 inhibitor. Western Blot analysis revealed a substantial decrease in NICD and RBPJ expression levels. These results suggested that the deacetylation activity of HDAC10 might influence the Notch-1 signaling pathway activity.

PTEN gene inactivation has been observed in various tumor types, including prostate, breast, and lung cancers, leading to decreased PTEN expression [38]. The Notch-1 pathway has also regulated PTEN expression in T-ALL and prostate cancer [39, 40]. In the present study, silencing HDAC10 in ccRCC cells significantly upregulated PTEN expression levels. Treatment with the HDAC10 inhibitor Quisinostat also led to a substantial upregulation of PTEN expression. These observations suggested that HDAC10's acetylase activity played a crucial role in regulating the expression of PTEN, a critical downstream target of the Notch-1 signaling pathway. Subsequently, we employed the Notch-1 signaling pathway inhibitor FLI-06 in ccRCC cells with depleted HDAC10 expression. Interestingly, upon inhibiting the Notch-1 pathway, we observed a further increase in PTEN expression levels compared to the control group, as validated by Western blot analysis. These results were consistent with observations in other types of tumors, suggesting that HDAC10 likely regulated PTEN expression, thereby affecting the progression of ccRCC through the modulation of Notch-1 pathway activation.

## Conclusion

Our study reveals a significant association between upregulated HDAC10 expression and advanced metastasis and an unfavorable prognosis in patients with ccRCC. Inhibition of HDAC10 substantially decreases cell proliferation and migratory capacity. Significantly, HDAC10 promotes tumorigenesis by activating the Notch-1 pathway, resulting in the downregulation of PTEN expression. In conclusion, our results indicate that HDAC10 can serve as a valuable prognostic biomarker in ccRCC patients, providing a potential therapeutic measure for the treatment of ccRCC.

### Supplementary Information

Additional file 1.

## Data Availability

The datasets used and/or analyzed during the current study are available from the corresponding author upon reasonable request.
