# Supplementary material for: Elevated histone deacetylase 10 expression promotes the progression of clear cell renal cell carcinoma by Notch-1-PTEN signaling axis
Source: Discov Oncol. 2024 May 11;15:156. doi: 10.1007/s12672-024-01018-9 (PMC11088579; doi:10.1007/s12672-024-01018-9)
Supplement: Supplementary file 1 — Additional file 1. [file 12672_2024_1018_MOESM1_ESM.docx]

**Elevated histone deacetylase 10 expression promotes the progression of clear cell renal cell carcinoma by Notch-1-PTEN signaling axis**

Bin Zheng^1†^, Xue Jiang^2†^, Yaqing Liu^3^, Fajuan Cheng^3^, Yiming Zhang^2^, Chengtao Niu^1^, Zixiang Cong^1^, Zhihong Niu^2^, Wei He^2*^

Corresponding author:

Wei He

Email: hewei@bjmu.edu.cn

Associate Professor

Department of Urology

Shandong Provincial Hospital Affiliated to Shandong First Medical University

**SUPPLEMENTARY MATERIALS**

**Supplementary Figures**

**
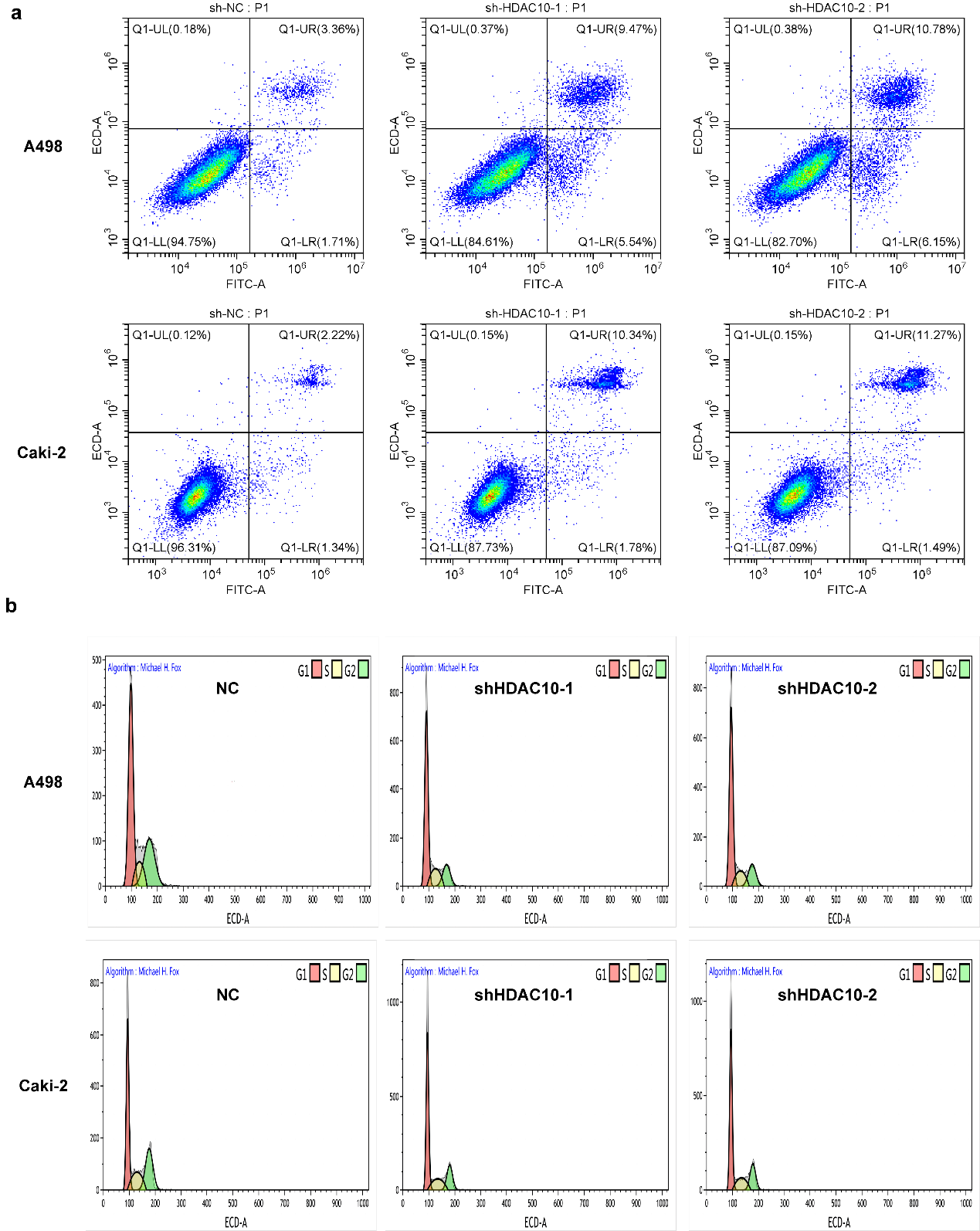
 Supplementary Fig.1**

**Effects of knockdown of HDAC10 on cell cycle and apoptosis** (a) Increased apoptosis was observed when treated with shHDAC10. (b) shHDAC10 significantly increased G1/S phase cells. (**P<0.01; ***P<0.001, ****P<0.0001).

**
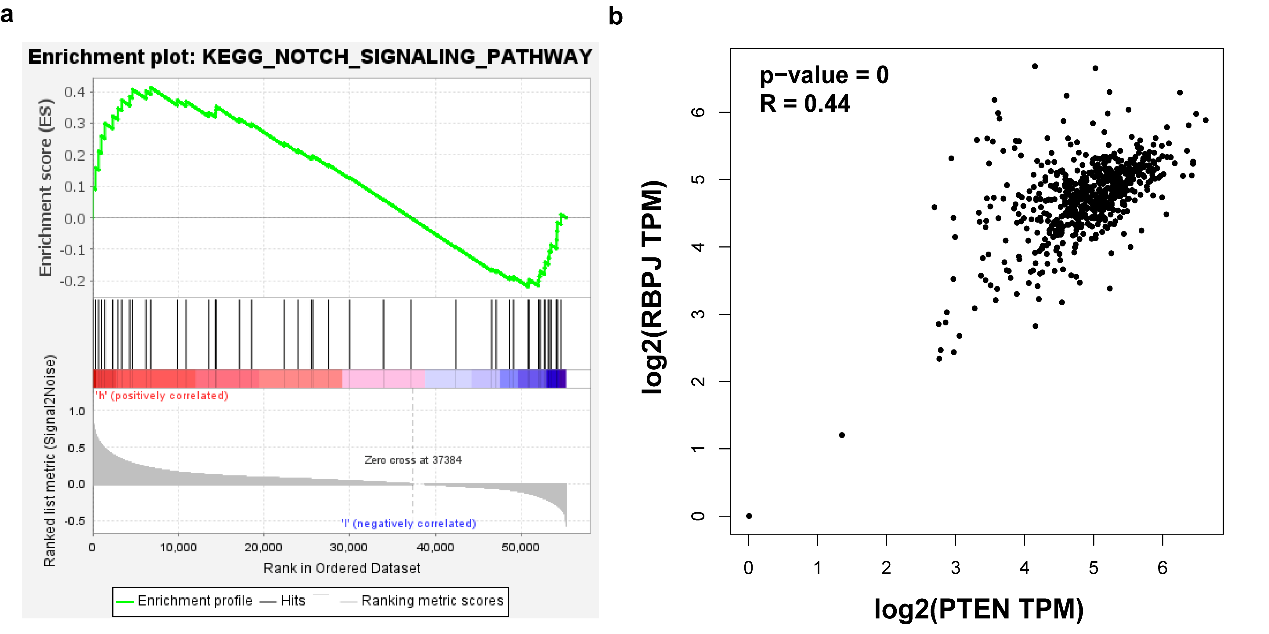
 Supplementary Fig.2**

**Pathways involved in the pathogenesis of HDAC10 with GSEA and correlation analysis between PTEN and RBPJ** (a) GSEA was conducted to examine the relationship between HDAC10 and the positive regulation of the Notch pathway. b) Correlation analysis using the GEPIA database was performed to determine the association between PTEN and RBPJ. (*p < 0.05, **p < 0.01, ***p < 0.001). GSEA: gene set enrichment analysis; GEPIA: Gene Expression Profiling Interactive Analysis.

**Supplementary Table**

**Supplementary Table 1 Primers sequences**

| HDAC10-F | TTCCTGCGAGAGTCAGATGC |
| --- | --- |
| HDAC10-R | TTTGCCCCTCAAAGGCCAGT |
| GAPDH-F | GTGGATATTGTTGCCATCAATGACC |
| GAPDH-R | GCCCCAGCCTTCTTCATGGTGGT |

**Figure 1-c GAPDH**

**

**

**Figure 1-c HDAC10**

**

**

**Figure 2-a A498 actin**

**

**

**Figure 2-a A498 HDAC10**

**

**

**Figure 2-a Caki-2 actin**

**

**

**Figure 2-a Caki-2 HDAC10**

**

**

**Figure 4-a A498 actin+NICD+RBPJ+PTEN**

**

**

**

**

**

**

**

**

**Figure 4-a Caki-2 actin+NICD+RBPJ+PTEN**

**

**

**

**

**

**

**

**

**Figure 4-b A498 actin+NICD+RBPJ+PTEN**

**

**

**

**

**

**

**

**

**Figure 4-b Caki-2 actin+NICD+RBPJ+PTEN**

**

**

**

**

**

**

**

**
